# Iodide of Potassium in Atonic Ulcers

**Published:** 1843-04

**Authors:** 


*Iodide of Potassium in Atonic Ulcers.*—The utility of the hydriodate of potass in secondary syphilis is pretty generally acknowledged. Lisfranc has employed it with success in ulcers of long standing, which have resisted other modes of treatment. A man, sixty-eight years of age, was admitted under his care at the Hôpital de la Pitie, who had for eight years had two obstinate atonic ulcers on his left leg; one of which, five inches in length, extended round the limb for more than half of its circumference; the other was about two inches in diameter. The only topical application used was simple cerate and charpie, but Lisfranc prescribed a scruple of the iodide of potassium daily, which quantity he subsequently increased to six grains every six hours. At the end of six weeks the health of the patient had become greatly improved; his flesh generally had acquired firmness; his face was ruddy; and he had even gained fat. The smaller of the two ulcers had completely disappeared in twenty-five days, and scarcely one-tenth of the larger ulcer remained to be healed. In two months the man left the hospital perfectly cured.—*London Lancet*, Jan. 7, 1843.

